# Diagnosing penicillin allergy in the absence of minor determinant mixture

**DOI:** 10.1186/1710-1492-10-S2-A43

**Published:** 2014-12-18

**Authors:** Lana Rosenfield, Chrystyna Kalicinsky, Richard Warrington

**Affiliations:** 1 Department of Internal Medicine, University of Manitoba, Winnipeg, Manitoba, Canada; 2 Section of Allergy and Clinical Immunology, Department of Internal Medicine, University of Manitoba, Winnipeg, Manitoba, Canada

## Background

Penicillin allergy is a common presentation in allergy clinic. The diagnosis of immediate hypersensitivity is made using clinical history, skin testing, specific IgE levels and oral challenge. Skin testing is done using benzyl penicilloyl-polylysine (PPL) and a minor determinant mixture (MDM) consisting of penicillin byproducts. Although using PPL and MDM is considered first line for diagnosis [1], our clinic is unable to consistently obtain MDM. We have undertaken a retrospective chart review to assess our current protocol in diagnosing penicillin allergy using Penicillin G (PG) alone instead of MDM.

## Methods

Charts of patients presenting to Allergy clinic with penicillin allergy between December 2005-Dec 2013 were reviewed. Skin testing by intradermal and skin prick testing was done using PPL and MDM or PG in addition to other possible suspected antibiotics. Patients who were negative for specific IgE testing to penicillin V and G (Immunocap) and had negative skin tests had oral challenge to penicillin.

## Results

Of 520 charts reviewed, 240 patients met criteria for analysis. 18 out of 240 patients had positive skin testing, eight to PPL, five to PG, four to MDM, six to ampicillin and one to ceftriaxone. Three patients had positive specific IgE levels with negative skin tests to PPL and PG. 222 patients underwent challenge. 88/222 had been skin tested to MDM and 8/88 had a positive challenge. 134/222 had been skin tested to PG and 4/134 had a positive challenge.

## Conclusion

9% of patients with positive oral challenge had negative testing with MDM compared to 3% with PG. Therefore we conclude that our current protocol is appropriate in diagnosing patients with penicillin allergy.
